# Embracing bilingualism in healthcare education: key stakeholders’ voices

**DOI:** 10.3389/fmed.2026.1747322

**Published:** 2026-03-09

**Authors:** Khatmah Alanazi

**Affiliations:** English Language Institute, Imam Mohammad Ibn Saud Islamic University (IMSIU), Riyadh, Saudi Arabia

**Keywords:** English medium of instruction (EMI), medium of instruction (MoI), healthcare education, bilingual education, stakeholders perceptions

## Abstract

**Introduction:**

In recent decades, higher education institutions worldwide have increasingly adopted English as the Medium of Instruction (EMI) across various disciplines, including medicine, to promote internationalisation and enhance access to global knowledge and markets. While EMI facilitates access to international medical knowledge and improves graduates’ global competitiveness, it also introduces linguistic and cognitive challenges, particularly in non-Anglophone contexts such as Saudi Arabia. This study aims to explore stakeholders’ perceptions of the medium of instruction in three medical programmes in a non-Anglophone country, Saudi Arabia.

**Methods:**

A mixed-methods approach was employed, integrating data from questionnaires, interviews, and classroom observations. Participants included a broad range of stakeholders—students, faculty members, and administrators—from three medical programmes in Saudi Arabia. Quantitative questionnaire data were used to identify general trends in perceptions toward EMI, while qualitative interviews and observations provided deeper insights into participants’ experiences and contextualized perspectives on language use in medical education.

**Results:**

Findings revealed that the majority of stakeholders supported EMI, citing its role in enhancing knowledge competency, professional preparedness, and English language proficiency. Despite this support, notable concerns emerged regarding content comprehension difficulties, increased cognitive load, and time constraints during teaching and learning processes. A recurring theme across data sources was the advocacy for a bilingual approach, whereby both English and students’ first language (Arabic) would be strategically employed to facilitate understanding, improve learning efficiency, and strengthen conceptual knowledge in both languages.

**Discussion:**

The results highlight the complex balance between globalisation goals and local linguistic realities in medical education. EMI is widely valued for its alignment with global medical standards and professional communication demands; however, its exclusive use may hinder learning outcomes for students with limited English proficiency. The strong endorsement of a context-sensitive bilingual approach underscores the need to integrate students’ first language and language support mechanisms within EMI policies. These insights provide actionable recommendations for policymakers, curriculum designers, and educators to optimise EMI implementation while maintaining educational equity and quality.

## 1 Introduction

Higher education institutions worldwide have adopted English as the medium of instruction (EMI) to boost internationalisation (e.g., attracting diverse students), employability (e.g., access to global job markets), marketisation of higher education (e.g., increased tuition revenue), and English proficiency (1, 2). EMI is defined as “the use of the English language to teach academic subjects (other than English itself) in countries or jurisdictions where the first language of the majority of the population is not English” [(3), p. 18]. EMI initial growth accelerated in the early 2000s in Europe [e.g., Spain (4), Italy (5)], followed by Asia [e.g., China (6), Vietnam (7)], and the Middle East [UAE, Saudi Arabia (3)].

However, despite its advantages, EMI presents a range of linguistic challenges that threaten content comprehension and the status of national languages. In non-Anglophone medical contexts where almost all institutions adopt EMI, English proficiency gaps hinder knowledge mastery and doctor-patient communication (8, 9). These gaps generate language barriers, cognitive overload, poor performance, and ultimately reduced healthcare quality (10–13). Researchers thus advocate L1 integration alongside English in bilingual EMI to facilitate comprehension and content learning (14, 15). While multilingualism is often regarded as a valuable resource for broadening communication and reducing cognitive load, it is frequently perceived as a barrier rather than a leverageable resource, thereby supporting monolingual approaches fuelled by the ideological prestige of English (16, 17).

In global non-Anglophone settings, including Arab contexts, English holds ideological prestige as linguistic capital for scientific knowledge, careers, and mobility (18, 19). This view frames Arabic as inadequate for modern medicine and science, associating it more with the humanities and classical texts, and ignoring its medieval role as a lingua franca (18, 20). EMI dominance thus reflects neoliberal rationales linking English to professional success, marginalising Arabic in academic discourse and reinforcing unequal linguistic prestige (21).

In Arab contexts, English-only medical education policies reinforce perceptions that local languages hinder English acquisition- a key EMI goal. However, EMI monolingualism overlooks students’ existing multilingual resources, which enable equitable learning by leveraging prior knowledge and creating psychologically safe classroom spaces (15). In many medical classrooms, low-proficiency students face extraneous cognitive load from acquiring new vocabulary and complex concepts delivered entirely in unfamiliar English (12, 22). Such EMI-induced extraneous load is especially pronounced in Arab medical curricula, where students shift from Arabic-medium schooling to English-dominant university programmes amid Arabic-reliant clinical practice (10). This generates excessive extraneous cognitive load, diverting students’ mental resources from essential medical processing to concurrent language decoding. Consequently, it heightens anxiety, disengagement, and policy tensions while impairing medical knowledge acquisition (11). These challenges become more pressing when considering cognitive load theory (CLT), which emphasises working memory limitations in processing new information (23). In EMI medical classes, instructional design fails to adequately account for students’ prior knowledge or linguistic backgrounds, fostering disengagement and anxiety (24, 25).

This situation proves especially severe in Arab medical education, where English exposure occurs mainly in formal university settings but rarely extends to Arabic-dominant clinical environments with patients and professionals (25). Bilingual EMI models address this study’s identified comprehension and clinical gaps by reframing multilingualism as an asset rather than a deficit, with translanguaging enabling strategic use of Arabic (L1) alongside English in Saudi medical programmes where proficiency varies (26, 27). Translanguaging deploys students’ full linguistic repertoires to create interactive spaces that scaffold concepts and reduce cognitive load (28–32). These approaches enhance accessibility and clinical outcomes, as evidenced by mixed-methods findings on MoI preferences, without compromising English proficiency goals (10, 22). Translanguaging theory interprets these as dynamic repertoire activation that optimises working memory under CLT (28, 33). Drawing on these theoretical insights and empirical findings, this study, grounded in CLT and multilingualism, examines stakeholders’ MoI preferences and motives in Saudi medical education to address perception gaps. It advances EMI theory and practice by integrating CLT with a stakeholder-motives-and-tensions framework for Arabic-supplemented instruction, with implications for professional preparedness and clinical communication.

### 1.1 Conceptual framework

To ensure conceptual clarity, the following key terms are defined consistently throughout the paper: Professional preparedness denotes bilingual readiness for patient communication, integrating Arabic (L1) and English resources to ensure diagnostic accuracy and patient safety. Knowledge competency requires mastery of medical content in lecture comprehension, conceptual clarification, and clinical application. The bilingual model involves strategic Arabic (L1) translanguaging within compulsory EMI to reduce cognitive load while maintaining exposure to English. Finally, Arabic-supplemented EMI refers to English-medium instruction systematically supported by targeted Arabic explanations and glossaries to optimize content mastery.

Situated in Saudi medical education, where English functions as a foreign language (EFL), this mixed-methods study examines stakeholders’ preferences for English-only versus Arabic-supplemented Medium of Instruction (MoI) and their underlying motives, addressing gaps in perceptions of EMI. Grounded in CLT (23) and extended by bilingual models, CLT posits that EMI imposes extraneous cognitive load from simultaneous language-content processing, which can be reduced by using strategic Arabic L1 to optimize germane medical learning. Effective learning further depends on managing three types of cognitive load—intrinsic, extraneous, and germane—within the limited capacity of working memory (23). Intrinsic load arises from complex medical terminology, driving a preference for Arabic-supplemented EMI to help manage comprehension difficulties. Extraneous load from EMI’s dual decoding motivates efforts to reduce language barriers to enhance comprehension. Germane load, essential for building clinical schemas, favors a bilingual model that supports progressive mastery and professional readiness. CLT proves particularly relevant for high-interactivity tasks in medical education, which integrate knowledge, skills, and behaviors that often exceed novices’ working memory capacity (34).

From a CLT perspective, translanguaging effectively manages intrinsic and extraneous cognitive loads in bilingual EMI by scaffolding new content in students’ stronger first language (L1). This process frees working memory for germane schema construction. Translanguaging involves the flexible use of multiple languages as an integrated repertoire for teaching and learning (28), creating spaces where languages coexist and interact to support each other (29, 30). This enables cross-linguistic connections, concept scaffolding, and cognitive load reduction. Recent studies confirm the benefits of translanguaging in EMI medical contexts for load management, content learning, and meaning making (31, 32). Drawing on CLT and multilingualism, this study examines medical stakeholders’ preferences and motivations regarding the medium of instruction, aiming to address gaps in perception by employing a mixed-methods approach to connect stakeholder views on EMI policy with classroom realities. It offers pedagogical insights for implementing EMI in Saudi medical education and contributes broadly to EMI research by analysing compulsory EMI in Saudi medical settings. It highlights how bilingual practices provide systematic support through the integration of CLT with dynamic bilingualism, demonstrating that students’ use of their first language (L1) mitigates EMI-related cognitive strain. Additionally, the study proposes cognitive load and bilingual models to address stakeholder tensions, ultimately enhancing clinical communication competencies in EMI contexts and serving as a valuable resource beyond a national case study. Based on the findings, practical recommendations are provided for EMI lecturers, curriculum developers, and institutional leaders, informing pedagogical strategies and language planning to overcome academic challenges in Saudi medical education and similar EMI contexts.

## 2 Literature review

### 2.1 EMI in Saudi Arabian higher education

In SA, Arabic is the official language and the most commonly used language in society, while English has long served as MoI in medical education due to its status as the universal language of medicine and science. In recent decades, Saudi medical education has undergone significant transformations, adapting to technological advancements to meet the needs of the healthcare sector. In 2000, a new phase began, driving the system toward excellence and recognition by international agencies, and to establish a self-sufficient workforce and increase the ratio of Saudi physicians (35). Consequently, many new medical schools have been established by the Saudi government, offering free education to Saudi students (36). Admission is based on high school GPA (90% and above), General Aptitude Test (GAT), and Scientific Academic Achievement Test (SAAT) scores (more than 85% on both), ensuring a high-ability cohort across the Bachelor of Medicine, Bachelor of Surgery (MBBS) years. Graduates’ and foreign professionals’ readiness to practice medicine is assessed by the Saudi Medical Licensure Examination (SMLE), which is conducted in English (37). To support these educational changes, preparatory year programs have been established in all Saudi universities, providing extensive English instruction for about 20 h per week over two semesters (38). In addition, hundreds of international English language instructors and content specialists have been hired from various countries, including Egypt, Morocco, India, Pakistan, the USA, the UK, and Canada.

These dynamics are particularly significant in Arab medical education, where students transition from Arabic-medium schooling to EMI university programs while predominantly using Arabic in healthcare encounters. This situation reveals a tension between EMI policies and clinical realities. The shift from Arabic-medium high schools to English-medium programs creates significant challenges. Despite 1 year of preparatory study, students’ limited English proficiency increases their cognitive load, hindering content comprehension, learning processes, and patient communication (10, 25). To mitigate these linguistic barriers and facilitate understanding, instructors commonly supplement their teaching with Arabic explanations (31, 39, 40).

### 2.2 Stakeholders’ perceptions of MoI

Research into EMI focuses on stakeholders’ perceptions of the MoI in higher education (HE). Findings reveal mixed views on EMI, heavily influenced by factors such as career prospects, global knowledge access, local contexts, and practical considerations (6, 41). However, there is a consistent positive perception of EMI over national languages. Many content lecturers support EMI policies for subject matter teaching, viewing English as a gateway to job opportunities and international mobility (42, 43). For instance, (44) investigation of 234 EMI lecturers in Afghanistan found strong support for EMI as it provides students with access to global knowledge, prepares them for professional careers, and helps them compete in the global job market.

Recent research has also focused on student perceptions of the MoI in HE [e.g., (45, 46)]. Many students express a preference for EMI, believing it enhances their English proficiency, career prospects, and confidence in participating in international events (47). Additionally, students view EMI as a pathway to global knowledge, as English is the dominant language in academic publishing and research (48, 49). For example, a study of a Thai EMI classroom (50) revealed that the majority of students strongly supported EMI for improving their English proficiency, which is crucial for accessing a wider range of resources and employment opportunities in both local and international job markets. These findings align with similar views on EMI globally, including in Bangladesh (51), South Korea (52), and China (53), where lecturers and students endorse EMI for career advancement and global access despite cognitive challenges.

### 2.3 Challenges of implementing EMI

Despite its advantages, such as global competitiveness and access to resources, EMI presents significant challenges for teachers and students in non-Anglophone countries. The literature reveals mixed views, with positive perceptions of EMI coexisting alongside concerns about knowledge competency and national language status. English-only policies lead to language difficulties, including challenges in understanding lectures, reading materials, and writing, primarily due to students limited English proficiency (54–57). For example, researchers indicate that Chinese students struggle with subject matter, writing, speaking, and engaging with materials, overwhelming their working memory and fostering anxiety and low participation (58, 59). These barriers inhibit learning, reduce comprehension, and limit accessibility, particularly in clinical training, where policies often prioritise globalisation over quality healthcare (7, 54). These findings align with evidence that foreign-language content study yields similar outcomes (60, 61).

Some studies highlight the need for English proficiency among multilingual patients, while others advocate for bilingual support (in both English and local languages, such as Arabic in Saudi Arabia and Spanish in Spain) to enhance local communication (10, 30). Such mismatches contribute to broader psychological and behavioral impacts in EMI settings, creating stress and anxiety among students with lower English proficiency due to difficulties in comprehending academic content and the pressure of peer competition (31, 62, 63). Additionally, EMI classrooms often experience lower levels of student participation and interaction (7, 63). In SA, for example, investigations have shown that EMI negatively impacts students by causing high levels of exhaustion and disengagement, reducing self-confidence, and increasing cognitive load (64). This echoes critiques of EMI regarding linguistic stress, equity gaps, and cultural differences as barriers to effective clinical training, which hinder patient communication (65).

Another significant concern is the impact of time constraints on cognitive resources for both teachers and students. Instructors working with low-proficiency students often find themselves dedicating extra time to explain concepts, which often leads to a sacrifice in both the depth and breadth of the content covered and the time available for student questions (66, 67). This situation increases extraneous cognitive load, diverting students’ working memory away from building effective medical schemas to inefficient decoding, exacerbating overload in EFL EMI classrooms.

### 2.4 Strategies for enhancing content comprehension

To address linguistic and cognitive barriers in EMI contexts, instructors employ strategies such as L1 simplification, repetition, translation, gestures, visuals, and models to help students navigate language barriers (5, 66). Students use their first language (L1) for better comprehension, which lessens cognitive load when encountering complex English medical terms. For instance, Arabic L1 strategies, such as glosses for terms like “Zira’at al-A’da” (organ transplantation), support understanding while still promoting English language learning (68). These approaches align with CLT by reducing extraneous cognitive load and targeting intrinsic load related to complex terminology and interactive elements (23). Furthermore, “instructors integrate students’ L1” alongside English through planned translanguage [e.g., L1 is used for explanations, (40)]. This integration enhances students’ learning experiences and effectively scaffolds their L1-English repertoires. Evidence from Saudi Arabia indicates that these L1 strategies, which include clarifying lectures with approximately 30% Arabic use and providing multimodal support, can enhance content retention by 25% without hindering English proficiency (26). Additionally, these approaches help lower anxiety by promoting collective comprehension (69). Global cases from Asia, including Pakistan (70), Vietnam (7), Bangladesh (51), and Spain (71), further confirm that L1 strategies enhance content comprehension and ensure equitable access in EMI contexts.

### 2.5 EMI in medical education and healthcare settings

Exclusive EMI in medical education imposes a high extraneous cognitive load on non-native English speakers due to the need to decode unfamiliar terms, which limits working memory capacity for intrinsic medical content and germane schema building, ultimately impairing bilingual proficiency essential for patient communication. Language barriers can lead to misunderstandings, resulting in misdiagnosis, inappropriate treatments, and decreased satisfaction for both patients and provider (72, 73). Research shows that patients with limited proficiency in the primary language spoken by healthcare providers are more likely to experience misunderstandings and less likely to adhere to medical advice (74). English-only EMI classes overload working memory with language processing, reducing comprehension and retention for non-native English students, while bilingualism through translanguaging alleviates this extraneous load by enabling rapid uptake of concepts via L1 glosses, as evidenced by Saudi studies where bilingual approaches improved post-test scores compared to English-only instruction (31).

This bridges to healthcare challenges: EMI barriers contribute to struggles in lectures and writing, leading to poor patient interactions and low satisfaction rates (72, 75). Equipping healthcare professionals with bilingual skills significantly reduces risks and improves outcomes, whereas reliance on interpreter services raises costs and poses issues related to training, misinterpretation, and confidentiality (76, 77). Studies indicate EMI-exclusive challenges in lectures, writing conventions, and material engagement, which impede performance and patient communication (12, 78). Research from North Africa highlights EMI proficiency gaps, with Tunisian students reporting poor outcomes without L1 support (79) and Algerian teachers noting performance limitations despite potential employability gains (80), revealing tensions between policy and practice. Similarly, in the Saudi healthcare context, EMI adds linguistic strain on top of medical content complexity, particularly where Arabic schooling precedes English-only lectures. Recent research indicates that medical students struggle with English-only lectures and academic writing, especially in the early years due to limited proficiency and short preparatory courses (10, 24, 81). These lead to cognitive overload, anxiety, lower performance, and burnout (64). For instance, a study (31) comparing first-year students’ biology comprehension in English-only versus bilingual classes found that bilingual students scored higher and reported less distraction and cognitive load. These findings underscore shared regional EMI challenges that create extraneous load for Arabic L1 students and support CLT-bilingualism frameworks, exposing misalignments between policy and practice.

In this respect, global EMI medical studies emphasise the role of students’ L1 in enhancing comprehension and patient care, with researchers advocating that local languages support medical content understanding and effective patient communication (9, 32). In Arab contexts, scholars assert that English as a scientific language should not overshadow the crucial role of the native language in non-Anglophone settings (13). They argue that bilingual instruction builds competency in both English and local languages to avoid miscommunication and compromised care. Additionally, a significant percentage of stakeholders, including students and instructors, support this approach, with 85% endorsing bilingual instruction for improved content comprehension (82). While teachers find L1 a time-saver for clarifying complex concepts for lower-level English students (24, 83), many medical students often translate terms and concepts into Arabic to aid understanding, which requires additional effort (81, 84, 85).

The literature shows that EMI in medical education has both short-term and long-term effects. In the short term, EMI can result in challenges such as language overload, comprehension difficulties, and miscommunication in clinical settings. However, it also offers long-term benefits, such as better access to global knowledge and improved career opportunities. Researchers advocate for bilingual approaches to enhance learning and support bilingualism. In this context, the study aims to explore stakeholders’ perspectives on the MoI in Saudi medical education through two targeted research questions:

1. What are stakeholders’ preferences for the MoI in Saudi Arabian medical education?
2. What factors influence stakeholders’ MoI preferences in Saudi Arabian medical education?

## 3 Materials and methods

### 3.1 Study context

The study was conducted at three newly founded (2006–2011) public medical schools in two major Saudi cities: Riyadh (Universities A and B) and Makkah (University C). These schools were purposively selected for high enrollment (200–300 students/year), flagship status, and EMI variation, including University B and the leading women’s University C in urban contexts. These capture stratified sampling of 34 content teachers, 17 English language instructors, and 289 students (Years 1–6), across gender-segregated EMI implementations. Representing 10% of Saudi Arabia’s 33 medical schools (30 public) and 20% of national students (86), they mirror curricula spanning lecture-based, problem-based learning (PBL), and community-oriented models under the National Center for Academic Accreditation and Evaluation, mandated EMI. Admission to these schools is based on a high school GPA of 90% and above, scores from the General Aptitude Test (GAT), and the Scientific Academic Achievement Test (SAAT), both requiring over 85%. The assessment methods include written components like portfolios and oral methods, such as the Objective Structured Clinical Examination (OSCE) (35).

### 3.2 Participants

Three participant groups were purposively sampled from three high-enrollment public medical schools (87). All participants were female due to recruitment from gender-segregated Saudi medical colleges and volunteer self-selection: Content teachers (CTs; *n* = 34), English language instructors (ELIs; *n* = 17), and Bachelor of Medicine, Bachelor of Surgery (MBBS) students, Years 1–6 (*n* = 289), total *n* = 340. This achieved representation across experience levels and bilingual backgrounds while respecting segregation norms. Recruitment targeted EMI faculty and students; response rates varied by institution from 24 to 67%, as shown in Table 1.

**Table 1:** CTs participant distribution and response rates by group and institution (*N* = 340; Res = response rate).

Institution	CTs n (Resp%)	ELIs n (Resp%)	MSs n (Resp%)
A	12 (58%)	7 (41%)	111 (37%)
B	9 (67%)	6 (35%)	80 (27%)
C	13 (31%)	4 (24%)	98 (33%)
Total	34	17	289
	340		

The first group is CT (*n* = 34), who taught medical courses using EMI with teaching experience spanning from 4 to 24 years. Of these, 12 (58%) from University A, 9 (67%) from B, and 13 (31%) from C (see Table 1). The CTs were aged 27–47 years and represented diverse nationalities: Egyptian (*n* = 9), Sudanese (*n* = 7), Saudi Arabian (*n* = 8), Indian (*n* = 3), UK (*n* = 2), Pakistani (*n* = 3), Filipino (*n* = 2). Twenty-nine were native Arabic speakers, and 5 had diverse L1 backgrounds (e.g., French, Urdu, Tamil, Filipino). The second group comprised 17 ELIs teaching English courses to medical students at preparatory-year programmes, with teaching experience ranging from 1 to 9 years. Of these, 7 (41%) were from Institution A, 6 (35%) from B, and 4 (24%) from C (Table 1). ELIs were aged 25–40 years and represented diverse linguistic backgrounds: 4 self-identified as native English speakers from countries including South Africa, UK, Canada, and USA. Meanwhile, 13 instructors spoke English as a second language with varied L1 backgrounds (e.g., Urdu, Arabic, French), highlighting the multilingual context relevant to EMI implementation.

The third group consisted of 289 Saudi students, aged 18–24 years, all native Arabic speakers. They represented six study levels, as illustrated in Table 2. By institution: University A (6-year programme) contributed 111 (38%) students, with quantitative responses (Yr1-2 *n* = 40, Yr3-4 *n* = 40, Yr5-6 *n* = 31) and qualitative interviews (*n* = 12, 8, 5). University B (4-year programme) contributed 80 (28%) students with quantitative responses (Yr1–2 *n* = 45, Yr3-4 *n* = 35) and qualitative interviews (*n* = 6, 8). University C (6-year programme) contributed 98 (34%) students with quantitative responses (Yr1–2 *n* = 37, Yr3–4 *n* = 32, Yr5–6 *n* = 29) and qualitative responses (*n* = 9, 7, 5). Across institutions by MBBS year: there were 122 students in Years 1–2, 107 in Years 3–4, and 60 in Years 5–6 (fewer due to final-year students now completing clinical rotations at affiliated hospitals).

**Table 2:** Distribution of student participants by institution and MBBS year.

Institutions	MBBS year	Part (n)	Res rate	Level of study	Quant (n)	Qual (n)	High school type
A	6 years	111	38%	Yr 1–2	40	7	Government (187; 65%)
A				Yr 3–4	40	5	
A				Yr 5–6	31	3	
B	4 years	80	28%	Yr 1–2	45	6	Private (62; 21%)
B				Yr 3–4	35	3	
C	6 years	98	34%	Yr 1–2	37	5	International (40; 14%)
C				Yr 3–4	32	4	
C				Yr 5–6	29	5	
Total		289	100%	Yr 1–6	289	38	289 (100%)

Quant, quantitative; Qual, qualitative; Part, participants.

Most students graduated from Saudi government schools (*n* = 187, 65%), followed by private (*n* = 62, 21%) and international schools in Saudi Arabia (*n* = 40, 14%). When asked to evaluate their English language proficiency, a majority (63%) rated themselves between “very good” and “excellent,” with others rating themselves as “good.”

### 3.3 Instruments and procedures

This study employed an explanatory sequential mixed methods design (88), collecting quantitative (through questionnaires) and qualitative data (interviews) concurrently to explain statistical findings through participant perspectives. Integration occurred at the interpretation stage, merging statistical results with emergent themes. Questionnaires were adapted from previous EMI studies with modifications for the Saudi medical education context and target participants. The student questionnaire was adapted from study (83), which examined EMI preferences and motives among students in Oman, a context similar to the current study, while the teacher questionnaire drew from study (89), which investigated non-English-proficient EMI teachers in Indonesia. The questionnaires contain three main sections: (1) demographic information (e.g., for students: age, language, school type, year of study; for teachers: age, nationality, language, years of experience); (2) participants’ preferences for the medium of instruction (three items: e.g., “English should be the MoI”, “Arabic should be the MoI”, and “English and Arabic should be the MoI”), and (3) the motives behind their preferences 18 items eliciting information on motives for EMI (e.g., “EMI provides access to medical knowledge”), motives for AMI (e.g., “Arabic helps me understand the content better”), and motives for bilingual instruction (e.g., “Using Arabic in EMI classrooms facilitates interaction with teachers). Both questionnaires used a 5-point Likert scale (Strongly Disagree, Disagree, Partially Agree, Agree, Strongly Agree) and (Never, Rarely, Sometimes, Often, Always) (87). No factor analysis was conducted on the adapted scale; construct validity was supported through triangulation with interview data. During analysis, responses were dichotomised (e.g., Strongly Disagree and Disagree = Disagreement; Partially Agree, Agree, and Strongly Agree = Agreement; Never and Rarely = Rarely; Sometimes, Often, and Always = Commonly) to simplify interpretation and enhance clarity for directional effects in medical education research [e.g., Arabic-supplemented EMI reduces cognitive load: yes/no, rather than how strongly they agree (90)].

The questionnaires were initially in English; however, to avoid misunderstandings related to English proficiency (91), the students’ questionnaire was translated into Arabic. Forward translation was conducted independently by two bilingual Arabic-English experts (one medical educator, one applied linguist). Discrepancies were resolved through discussion with a third bilingual expert, ensuring semantic and cultural equivalence through content review. Arabic versions were piloted with 7 medical students from the targeted schools (3 Year 1, 2 Year 3, 2 Year 5) and modified based on feedback from think-aloud sessions and comprehension checks, such as reducing the number of repeated items from 21 to 18, and wording changes (e.g., “EMI facilitates access to global knowledge” changed to “English gives access to medical materials”). Reliability was ensured through Cronbach’s alpha (92), which exceeded 0.90 (0.92 for teachers, 0.94 for students). The questionnaires were subsequently distributed to participants on their campuses, allowing them to complete them anonymously to protect their privacy (93). Ethical approval was obtained before data collection. Participants provided informed consent (written or oral), ensuring understanding of study aims and data confidentiality. Oral consent accommodated participants’ cultural preferences for verbal agreements while ensuring ethical compliance. An invitation to the next stage (interview) was added to the end of the questionnaire. Following this stage, the researcher organised times to conduct interviews with participants who had agreed to take part.

The semi-structured interviews were conducted individually with 60 purposively selected participants (15 CTs, 5 ELIs, 2 deans, and 38 MBBS students across years 1–6, and 2 deans) at their institutions (Table 3). This ensured variation in schools, age groups, teaching experience, and teacher nationalities (e.g., Arab, Asian). Questions derived from questionnaire domains used standardised probing prompts (e.g., “Can you elaborate on why…?”) to elicit elaborated responses (94). To encourage participants to express their thoughts freely, the questions were designed with three open-ended sections: background, MoI preferences, and motives (95).

**Table 3:** Interview sample overview.

Participant group	Number	Description
CTs (content teachers)	15	Selected for institution and age diversity with varied teaching experience and nationalities (e.g., Arab, Asian).
ELIs (English language instructors)	5	
MBBS students	38	Years 1–6 from different schools.
Deans	2	Institutional leaders.
Total	60	

For Arabic-speaking participants, the English guide underwent rigorous forward-backward translation (96). A native Arabic expert (MA in Translation Studies) forward-translated it into Arabic. A second expert (native English speaker fluent in Arabic) independently back-translated it into English. The researcher and both experts then collaboratively reviewed the original English, Arabic, and back-translated versions, resolving discrepancies for semantic and conceptual equivalence. Revisions occurred iteratively across two rounds. For linguistic accuracy (e.g., “cognitive load” became “al-‘ib’ al-ma ‘rifī”) to match precise MBBS terminology. For cultural appropriateness, the directive phrase like ‘Why did you struggle?’ was rephrased to ‘What challenges did you face?’. Following finalisation, a pilot interview with two allied medical students provided feedback that prompted minor changes to the wording and timing (e.g., “Tell me why” changed to “What are your thoughts?”). These steps enhanced transparency, minimised errors, and ensured reliability across languages (97). Interviews were conducted individually at participants’ schools to ensure contextual authenticity and were audio-recorded with permission for accurate verbatim transcription and thematic analysis. The sessions lasted between 7 and 15 min due to institutional time constraints and the scheduling of participants’ classes. While short durations may limit data depth and richness, this was mitigated by thematic saturation across 60 interviews and triangulation with questionnaire data (*n* = 340).

### 3.4 Data analysis

Quantitative data were analysed using SPSS Statistics (version 25.0). Descriptive statistics (frequencies and percentages) characterised cognitive load and MoI preferences across groups, years, and institutions. Deductive coding was applied through a bilingual lens, viewing Arabic L1 as part of the repertoire, which enhanced EMI comprehension and facilitated the theoretical interpretation of stakeholder perceptions. Interview data underwent reflexive thematic analysis (98), conducted as a hybrid inductive-deductive approach (99). Utterances in Arabic were first transcribed verbatim immediately after each interview, focusing on the main thematic content while excluding pauses, fillers, and repetitions. Arabic portions were forward-translated into English, and then independently back-translated by a second bilingual expert (native English speaker fluent in Arabic) with discrepancies resolved via collaborative discussion [see (96)]. NVivo facilitated data organisation and systematic inductive open coding of emergent patterns from common words and phrases (e.g., “English is important”). This was followed by deductive coding aligned with EMI literature and questionnaire scales. The transcripts were independently analysed by two coders (the researcher and an independent coder) to ensure reliability and reduce bias, achieving high intercoder agreement through iterative discussions (100).

To maintain anonymity and privacy, unique codes were assigned to all participants (CTs, ELIs, MSs) while contextualising perspectives by stakeholder group. For example, CTs use the first three letters of their name followed by CT and years of experience in EMI (e.g., Sar-CT, 6 yr. EMI); ELIs use the first three letters followed by ELI and years of experience (e.g., Ran-ELI, 3 yrs); and students, the largest group (38 MSs), were presented with (S), followed by a number and the level of their study (e.g., S2, Y5). Trustworthiness was ensured through member checking (i.e., themes verified with 7 participants), coder agreement, and triangulation of interview results with quantitative patterns. Key themes were presented in joint display tables to illustrate quantitative agreements or discrepancies, such as the 55% preference for BA among MSs in the survey elaborated by interviews citing challenges in EMI [see (101)].

## 4 Findings

The analysis of the questionnaire and interview data identified two primary themes: (1) support for EMI and (2) concerns about the exclusive use of English, along with support for Arabic-supplemented EMI. These themes are discussed in the following sections in relation to the literature review.

### 4.1 Support for EMI

**Figure 1:**
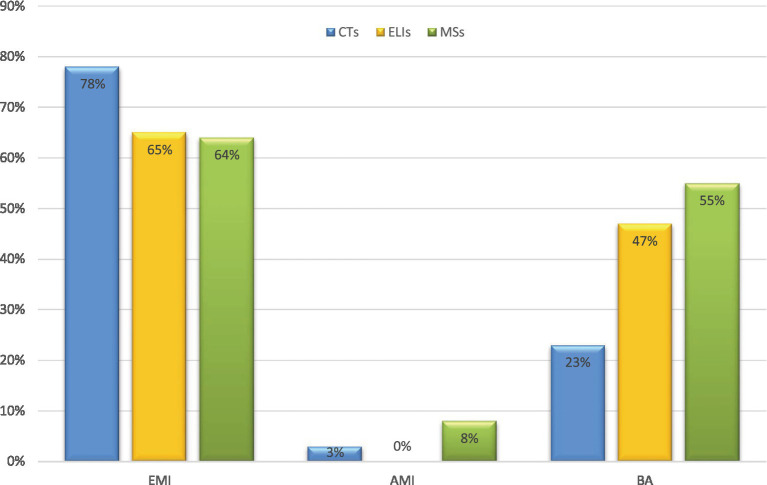
Stakeholders’ preferred teaching languages. Multiple responses permitted (EMI, English Medium Instruction; AMI, Arabic Medium Instruction; BA, Bilingual Approach). ELIs (*n* = 17), CTs (*n* = 34), MSs (*n* = 289).

The findings from the questionnaire responses to RQ1 indicate a strong preference among participants for the exclusive use of English in medical education. Specifically, the majority agreed with the statement, “It is better to teach medicine using English-only.” (65% of ELIs, 78% of CTs, 64% of MSs). A substantial proportion preferred “teaching medicine using both Arabic and English”, (47% of ELLs, 23% of CTs, and 55% of MSs, and only a small minority endorsed “teaching medicine using Arabic only), (0% of ELLs, 3% of CTs, and 8% of MSs), as illustrated in Figure 1.

To further clarify these results, ELIs, CTs, and MSs were interviewed. When comparing teaching medicine through EMI, AMI, or BA, the majority of participants expressed moderate-to-strong preference for EMI, citing three main motives: the status of English as a global language, opportunities for English language development, and enhanced professional preparedness. Together, these motives collectively address RQ2.

#### 4.1.1 The status of English as a global language

One of the main reasons cited across groups is the role of English as a global language of science. Participants explained that medicine should be taught in English because it is the international language of science. This choice, motivated by the belief that English provides access to global knowledge, facilitates communication with professionals and colleagues, and helps practitioners stay updated with the latest medical information:

"Medicine should be taught in English because it is the international language and the language of science" (Rem, CT, 9 yrs EMI).

"It is better to teach medicine in English so we can stay up to date with the latest medical information" (S7, Y5).

#### 4.1.2 Contributions of EMI to students’ English language development

The second reason cited by participants was the valuable role EMI plays in developing students’ English language proficiency. Although the primary goal of EMI is to teach medical content, most participants noted that EMI programmes are also driven by the need to develop students’ English proficiency. They reported improvements in students’ English skills needed for academic and workplace contexts, indicating that EMI enhances speaking (88% of both ELIs and CTs, 78% of MSs), listening (94% of ELIs, 85% of CTs, 78% of MSs), reading (94% of ELIs, 85% of CTs, 81% of MSs), and writing (94% of ELIs, 85% of CTs, 75% (*n* = 45) of MSs), as well as technical vocabulary (88% of ELIs, 85% of CTs, 86% of MSs; see Table 4).

**Table 4:** Perceived contributions of EMI to students’ English proficiency (% participants’ agreement).

Skills	ELIs (*n* = 17)	CTs (*n* = 34)	MSs (*n* = 289)
Writing	94% (16)	85% (29)	75% (217)
Speaking	88% (15)	88% (30)	78% (225)
Reading	94% (16)	85% (29)	80% (231)
Listening	94% (16)	85% (29)	78% (225)
Vocabulary	88% (15)	85% (29)	86% (249)

Through the interviews, ELIs and CTs observed noted how MSs rapidly gain English proficiency. Many students “enter the programme with a low or intermediate level of English”, but within “one or two years, they become capable of handling communication in various situations and comprehending reading materials” (Nad, CT, 11 years EMI; Afn, ELI, 6 years EMI). They perceive the EMI programme as “a bridge between high school and the university” that helps “build students’ language proficiency and confidence.” (Ran, CT, 7 years EMI).

Students also reported significant gains in their English skills from the EMI programmes, noting improvements from entry to their current levels in reading, listening, writing, and speaking. They explained that EMI “helps in improving [their] speaking and communication abilities… through reading, analysing, and discussing a medical case in a PBL setting” (S30-Y4), as well as their “reading and writing skills” (S10-Y5). However, some interviewed CTs believed that students still need more support to effectively develop their academic writing. They suggested that writing has been marginalised, despite its essential role in medical contexts, where students are required to write medical reports and research papers, as noted by Mar:

“Yes, EMI plays an essential role in improving most students' English language skills; however, it does not adequately address writing. Students still need to enhance their writing skills specifically for a medical context" (Mar, CT, 23 yrs EMI)

In addition to developing language skills, the interviews revealed that students’ vocabulary growth through EMI enhanced their communication skills, specifically in reading, speaking, writing, and understanding, and contributed to their academic achievement. For example, one student stated: “I have learned many academic words and technical terms, which have helped me understand complex texts and boosted my confidence in delivering clear presentations” (S6, Y3).

#### 4.1.3 Contribution of EMI to students’ professional preparedness

The third cited advantage of EMI is its contribution to students’ professional development and career preparedness. The analysis shows that the majority of participants highlighted the significant role of EMI in preparing students for future careers (76% of ELIs, 80% of CTs, 75% (*n* = 45) of MSs) by enhancing their English proficiency, which in turn facilitates the learning process (82% of ELIs, 80% of CTs, 70% of MSs), and develops their professional knowledge through improved access to medical information (88% of ELIs, 83% of CTs, 66% of MSs) (Table 5).

**Table 5:** Perceived contributions of EMI to professional preparedness.

EMI benefit	ELIs (*n* = 17)	CTs (*n* = 34)	MSs (*n* = 289)
Preparing for future career	76% (13)	80% (27)	75% (217)
Facilitating learning process	82% (14)	80% (27)	70% (203)
Providing access to information	88% (15)	83% (28)	66% (191)

In the interviews, participants reported that EMI enhances students’ language proficiency and develops their professional knowledge, demonstrating substantial growth in medical knowledge from the beginning to the end of the course. This includes “skills for academic writing, scientific reading”, as well as the “skills needed for effective communication with professionals at local and international levels” (S25, Y6). Teachers further explained that EMI-related activities, such as giving presentations, writing research papers, and participating in case discussions, strengthen students’ language abilities and foster professionalism through student-teacher interactions:

“In EMI medical programmes, students engage in various activities such as searching for information, reading, delivering presentations, writing research papers, and participating in class discussions. These activities collectively prepare them for both academic and professional life"(Gih, CT, 10 yrs EMI; Lyn, ELI, 9 yrs EMI).

The Dean of a foundation program supports EMI for teaching medicine in the Arab world, aiming to “provide students with access to up-to-date medical knowledge and prepare them for participation in specific international events such as conferences” (Dean-Abd).

Furthermore, although some participants suggested using Arabic for teaching medicine, there was near-universal agreement that reverting to Arabic as the MoI would currently be impossible. For instance, only 3% of CTs and 8% of MSs agreed to adopt AMI for teaching medicine. This sentiment was supported by three main reasons identified in the interviews: (1) “a lack of medical resources available in Arabic”(Man, CT, 26 years of EMI; Ree, ELI, 7 years; S1-Y3); (2) a “shortage of qualified teachers capable of teaching medicine entirely in Arabic “(Rab, Ama, CT, 13, 4 years, EMI; S17, S11-Y3,4); and (3) a lack of consistent equivalents for many medical terms in Arabic” (Abe, CT, 15 years of EMI, S9, S28-Y4).

Complementing these structural constraints, the quantitative endorsement of EMI’s role in career preparation (75–80%, Table 5) aligns with qualitative motives for global access. Notably, there is a disparity in perceptions regarding proficiency, as indicated by student perceptions (66%) compared to faculty perceptions (83–88%), reflecting proficiency gaps. Advanced MSs prioritise clinical Arabic post-mastery, stating: “Now in clinical rotations, I need Arabic…” (S13, Y6). This quantitative-qualitative pattern underscores the strengths of EMI while highlighting the need for L1 supplementation.

### 4.2 Support for Arabic-supplemented EMI

Despite strong quantitative support for EMI in medical teaching and learning (>60%), participants expressed concerns about the quality of English-only instruction. They believed that incorporating Arabic into EMI classrooms would enhance content learning, facilitate classroom interaction, and better prepare students for effective patient communication. Participants articulated these perceived benefits in three key areas, beginning with the role of Arabic supplementation in supporting content comprehension.

#### 4.2.1 Arabic-supplemented EMI supports content learning

When asked about EMI’s role in facilitating content understanding, only 45% (*n* = 130) of students agreed (Table 6), markedly lower than faculty (ELIs 53%; CTs 68%), indicating implementation challenges. Instructors and MSs’ responses also showed that students were less engaged in English-only classes; only 29% of ELIs, 37% of CTs, and 40% MSs agreed with “EMI makes topics more interesting”.

**Table 6:** Stakeholder agreement on EMI academic and clinical benefits (*n* = 340).

EMI advantage	ELIs (*n* = 17)	CTs (*n* = 34)	MSs (*n* = 289)
Facilitates understanding of content	53% (9)	68% (23)	45% (130)
Enhances interaction in classroom	29% (5)	32% (11)	48% (139)
Makes topics more interesting	29% (5)	37% (13)	40% (116)
Prepares for patient communication	59% (10)	35% (12)	24% (69)

Qualitative data elucidate this divergence, with first-year students reporting comprehension difficulties from English-only lectures due to non-equivalent terms and time-intensive mental translation [e.g., “I did not really understand most of the English-only lecture…” (S26, Y2); “it takes me hours…translating one lecture in my first semester” (S16, Y5)]. CTs corroborate the low 45% (*n* = 130) student agreement on EMI content understanding, noting that low-proficiency MSs experience language anxiety and disengagement in English-only classrooms, hindering interactive activities like case discussions.

The analysis also shows that Arabic-supplemented EMI leverages students’ full L1 repertoire for faster concept grasp, time-efficient coverage, and reduced home study time via translation. CTs confirmed this, stating that using Arabic “makes it easier for students to understand new concepts, facilitates content teaching, and promotes bilingualism” (Ran, Faw CT,16Y,13Y, EMI). Students also valued Arabic supplementation for enhancing content comprehension and reducing study time [e.g., “I enjoy classes where Arabic language is used alongside English… understand lectures quickly and save study time” (S21-Y1)]. This qualitative evidence explains quantitative patterns of ambivalence toward EMI: 47–55% of participants prefer bilingual approaches (Figure 1), with year 1 and 2 students reporting barriers (45%) despite faculty support (53–68%).

#### 4.2.2 Arabic-supplemented EMI facilitates classroom communication

When participants were asked if English-only EMI enhances teacher-student interaction (“using English only enhances interaction between teachers and students”), most disagreed (29% ELIs, 32% CTs, 48% MSs agreed; Table 6). Students reported frequent Arabic use in EMI classrooms for key activities: asking questions (31% commonly, 34% sometimes), answering teachers (32% commonly, 35% sometimes), participating in group discussions (36% commonly, 35% sometimes), providing local examples (e.g., disease names; 30% commonly, 34% sometimes), and sharing cultural values (56% commonly, 23% sometimes) (Table 7; *n* = 289 MSs). This shows students’ engagement in Arabic-supplemented EMI classes is higher due to greater, more active, and more interested student participation, compared to the 40% who enjoy English-only classes (Table 6).

**Table 7:** Frequency of Arabic use in EMI classroom activities (*n* = 289 MSs).

Classroom activity	Commonly	Sometimes	Rarely
Asking questions	31% (90)	34% (98)	35% (101)
Answering teachers’ questions	32% (92)	35% (101)	33% (96)
Participating in group discussions	36% (104)	35% (101)	29% (84)
Giving examples from local context	30% (87)	34% (98)	36% (104)
Sharing social or cultural values	56% (162)	23% (67)	21% (60)

In the interviews, some students indicated that they avoided English discussions and answering questions due to limited English proficiency and judgment fears:

“When my teacher uses Arabic during lectures, it signals that Arabic is acceptable. I feel less stressed. I sometimes ask questions in Arabic, and this ensures I fully understand the topic before leaving the class” (S4, Y1).

“In my first semester, I did not really enjoy group discussion where we must use English only. I felt I could not express my ideas freely.” (S4, Y2).

#### 4.2.3 Arabic-supplemented EMI prepares students for successful communication in healthcare

Further evidence supporting Arabic-supplemented EMI in the medical context is the general disagreement among CTs and MSs regarding EMI’s role in preparing students for meaningful communication with Arabic-speaking patients. The analysis further indicates that only 35% of CTs and 24% of MSs agreed that EMI adequately “prepares students for communication with Arabic-speaking patients”, contrasting ELIs’ optimism (59%, Table 6). This finding is reinforced by advanced clinical students (Years 5–6), who highlighted the growing importance of Arabic during clinical training:

“In early years, I relied solely on English for content understanding. Now in clinical rotations, I need Arabic to communicate with local patients. Sometimes, I struggle to explain concepts or name diseases in Arabic” (S13, Y6).

The analysis of qualitative and quantitative data shows strong support for EMI, particularly in terms of career preparation, access to medical knowledge, and the development of English proficiency. However, lower agreement regarding classroom interaction and preparation for communication with Arabic-speaking patients highlights persistent challenges, which interview data attribute to language anxiety and clinical communication gaps that can be addressed through Arabic supplementation.

## 5 Discussion

Overall, the findings demonstrate strong quantitative support for EMI (Tables 4, 5), with over 60% agreement regarding its role in career preparation (75–80%), access to information (66–88%), and English proficiency development in writing, speaking, and reading (75–94%). Qualitative data align with these results, framing EMI as an “international language” that provides “access to up-to-date medical knowledge,” thereby confirming its contribution to academic skill-building and global competitiveness. However, notable discordance appears (Table 6) in areas such as interaction enhancement (29–48%) and preparation for patient communication (24–35%). Interviews clarify this divergence, highlighting language-related anxiety and clinical communication gaps that can be addressed through Arabic supplementation. Together, these highlight three main findings: strong EMI endorsement, persistent implementation challenges, and growing advocacy for bilingual pedagogies.

First, the findings reveal stage-specific shifts in EMI attitudes across Saudi medical education, diverging from prior research that reports consistently positive EMI perceptions. While preclinical students (Years 1–3) largely prioritise English for accessing global medical knowledge (64%), this preference declines among clinical trainees (Years 4–6) who increasingly favour Arabic-supplemented EMI to meet patient communication demands (Figure 1). This shift is supported qualitatively, with all interviewed students (n = 38) endorsing bilingual approaches, mirroring a quantitative decline in EMI-only endorsement (65–55%). This pattern aligns with previous research reporting reduced preference for English-only instruction in clinically intensive contexts despite recognition of EMI’s academic value (13, 53, 54). These patterns expose persistent tensions between English-mediated academic access and Arabic clinical communication. They also underscore cognitive load concerns, supporting calls for bilingual instructional models (24, 25), Qualitative data further illuminate this tension: although preclinical students value English immersion for career preparation, they report substantial cognitive demands associated with language decoding (*n* = 28 excerpts). Together, these quantitative and qualitative findings highlight persistent tensions between English-mediated academic access and Arabic clinical communication, underscore cognitive load concerns, and exemplify EMI’s so-called “dark side” (65), whereby language demands intensify cognitive overload. Collectively, the evidence supports early Arabic scaffolding as a means to reduce cognitive strain while preserving EMI’s global advantages, challenging the monolingual myth prevalent among Arab stakeholders (102), and positioning Arabic as a strategic, rather than peripheral pedagogical resource.

Second, this tension extends to knowledge acquisition and professional development. Participants expressed concerns that exclusive English delivery diverts first-year students’ efforts from conceptual understanding to linguistic processing, such as translating lecture content, often resulting in disengagement (24, 25). This pattern reflects how English-only instruction imposes high extraneous cognitive load on Arabic L1 students by requiring continuous decoding, thereby depleting cognitive resources needed for germane processing and learning (23). Newly enrolled students are particularly vulnerable to overload due to underdeveloped disciplinary schemas and limited familiarity with medical terminology, which further strains working memory capacity (35). In this context, targeted L1 Arabic scaffolding can mitigate this strain, freeing capacity for content mastery (23).

Third, many student participants advocated for bilingual models, integrating L1 (Arabic) alongside EMI. They argued that exclusive English instruction impedes content comprehension, classroom interaction, and the development of doctor–patient communication skills, which are essential in Saudi healthcare. Similar concerns have been reported globally (10, 73, 103). Qualitative findings help explain the quantitative ambivalence: 47–55% of students prefer bilingual approaches (Figure 1), despite overall support for EMI. While instructors often prioritise EMI for career preparation, students report that overreliance on English can be mitigated by supplementing with L1 in Years 1–2, reflecting both faculty (53–68%) and student (45%) perspectives. This aligns with other Saudi EMI studies, which show that incorporating L1 enhances comprehension without limiting access to global knowledge (10, 25). These challenges can be understood through Cognitive Load Theory (CLT): English-only instruction imposes extraneous load, limiting germane schema construction for clinical proficiency. CLT-grounded bilingual scaffolding reduces linguistic strain, preserves global readiness, and enhances classroom and patient interaction. Although participants rejected full AMI due to scarce materials and absent standardised medical terminology (102, 104), they repositioned Arabic as EMI scaffolding, countering marginalisation while maintaining international access. Short-term constraints, such as textbooks, unqualified teachers, and English dominance, should not preclude hybrid AMI-EMI curricula, faculty bilingual training, and localized Arabic medical resources. This approach aligns with global evidence: Arab contexts [e.g., Tunisia and Algeria (79, 80)] and other EMI global contexts (Poland, China) (15, 75) report similar gaps in preparing students for healthcare skills, highlighting the need for bilingual models. L1 supplementation optimises germane load, facilitates concept transfer, eases anxiety, and strengthens classroom engagement. Thereby resolving policy-practice tensions without undermining EMI’s academic benefits (32, 70, 105). Together, these findings support CLT-grounded bilingual models that scientifically validate L1 (Arabic), promote equity in multilingual EMI, and foster dual proficiency in both English and Arabic, which is essential for the Saudi healthcare sector.

## 6 Conclusion and implications

Overall, participants in this study viewed EMI policy as an opportunity for language development, career advancement, and resource access, though tempered by cognitive load and clinical demands. Students, in particular, reported difficulties comprehending exclusively English content and noted inadequate training in patient communication skills. Thus, they endorsed Arabic-supplemented EMI to support both academic achievement and professional readiness. The findings suggest a broad consensus that, while English remains the primary MoI, incorporating Arabic enhances content comprehension and facilitates communication between Arabic-speaking doctors and patients. This pattern is consistent with bilingual education research showing that strategic L1 use supports content understanding, mitigates cognitive load, and better equips practitioners for local clinical practice, extending evidence from other Arab contexts (e.g., Tunisia, Algeria). Building on these findings and prior suggestions [e.g., (48)], three recommendations emerged to address cognitive load and clinical demands in medical education. First, students’ linguistic skills require holistic support for medical knowledge construction, professional readiness, and effective physician-patient communication. Second, an abridged English preparatory course should target EMI-specific medical skills for first-year students. Third, the findings underscore the crucial role of Arabic supplementation in enhancing EMI medical learning and improving physician-patient communication. This integration of Arabic not only facilitates better understanding of medical concepts but also helps future practitioners communicate more effectively with Arabic-speaking patients, thereby bridging the gap in healthcare delivery. Building on this, the study proposes a framework to optimise learning outcomes and reduce cognitive load through staged Arabic supplementation across MBBS years.

Table 8 operationalizes a CLT framework by mapping targeted bilingual EMI strategies to curricular stages. In Years 1–2 (pre-clinical), Arabic clarification targets complex medical terminology translation into Arabic during English lectures, freeing working memory for deep content processing in basic sciences like anatomy, physiology, and biochemistry, countering simultaneous decoding overload evidenced by 45% (*n* = 130) students (Table 6).

**Table 8:** Proposed Arabic-supplemented EMI by MBBS stage.

MBBS Year	Curricular Focus	Arabic-Supplemented Strategy	Cognitive Load Benefit
Year 1–2 (pre-clinical)	Basic sciences	English-only lectures and Arabic clarification	Reduces extraneous load during content learning
Year3–4 (clinical rotations)	Case discussions	Bilingual cases (Arabic history and English diagnostics)	Enhances germane load for clinical reasoning
Year 5–6 (internship)	Patient communication	Translanguaging (L1 rapport and English with professionals)	Safeguards patient safety

In Years 3–4 (clinical rotations), bilingual cases enhance germane load for clinical reasoning by minimising extraneous demands from English-only instruction (e.g., Arabic patient histories lower decoding load). This addresses 48% student-reported interaction barriers (Table 6) while boosting schema-building for differential diagnosis and treatment planning. Finally, in Years 5–6, translanguaging safeguards patient safety in high-stakes internships by utilising Arabic to build trust and obtain accurate patient histories, while English is reserved for precise diagnostics and medical testing. Arabic explanations reduce cognitive overload, particularly given only 24% student agreement that EMI prepares them for Arabic-speaking patient interactions (Table 6), while prioritising professional communication demands.

This staged CLT-bilingual framework bridges EMI policy-practice gaps in Saudi medical education. It aligns compulsory English with classroom realities (e.g., low proficiency, Arabic-dominant patient care) via targeted MBBS supplementation. This cuts extraneous load and boosts germane processing for clinical proficiency. Limited to female participants from three gender-segregated Saudi medical schools, the findings preliminarily suggest L1 strategies counter marginalisation and optimise learning efficiency in comparable EMI contexts. These findings have potential for global EMI application; however, they require validation across multiple, mixed-gender sites rather than a single national case study.

### 6.1 Limitations and future research

While this study offers valuable insights into EMI in Saudi medical education, it is important to consider certain context-driven factors that shape its findings. In particular, the focus on a female-only participant group, a reflection of institutional gender-segregation norms, limits the generalisability to male stakeholders, whose experiences with language confidence or cultural norms in different educational settings may vary. Furthermore, relying on self-reported perceptions, while useful for capturing attitudes, means the findings offer less direct insight into observable pedagogical effectiveness (e.g., specific classroom interactions or student outcomes). These considerations highlight the importance of future research expanding to mixed-gender settings and utilizing multi-method approaches, such as incorporating classroom observations or performance data, to further validate and enrich the understanding of MoI policies. Additionally, the short interview duration, although sufficient for achieving thematic saturation across 60 participants, could be complemented in future studies by longer interviews to add further depth.

## Data Availability

The datasets presented in this article are not readily available because the data can be seen by the researcher only. Requests to access the datasets should be directed to khtmhnahar@gmail.com.
